# Exploring the relationship between materialism, consumer ethnocentrism, and compulsive buying

**DOI:** 10.3389/fpsyg.2025.1680164

**Published:** 2025-11-10

**Authors:** Róbert Štefko, Martin Rigelský, Ivana Ondrijová, Lenka Kráľová

**Affiliations:** 1 Department of Marketing and International Trade, Faculty of Management and Business, University of Presov, Prešov, Slovakia; 2 Department of Managerial Psychology, Faculty of Management and Business, University of Presov, Prešov, Slovakia

**Keywords:** materialism, ethnocentrism, compulsive buying, purchasing behavior, consumer behavior

## Abstract

Materialistic values, consumer ethnocentrism, and compulsive buying represent three significant concepts in the field of consumer behavior. Theoretical models suggest that materialism may contribute to impulsive and compulsive purchasing behavior, while consumer ethnocentrism influences preferences for domestic products and attitudes toward foreign goods. The interconnection between these three constructs has been only marginally explored, especially in smaller Central European markets such as Slovakia, where cultural and socio-economic factors may shape consumer value orientations differently. The aim of this study was to analyze the relationships between materialistic values, consumer ethnocentrism, and compulsive buying among a sample of Slovak respondents. The research was based on data collected by the agency FOCUS through an online panel using quota sampling to ensure representativeness by demographic characteristics. The study also investigates whether ethnocentrism acts as a mediator between materialism and compulsive buying. Data was collected through an online questionnaire containing validated scales: the Material Values Scale, the Consumer Ethnocentrism Scale, and the Compulsive Buying Scale. To analyze the relationships between the variables, correlation analyses, linear regression, and mediation analysis using bootstrapping were employed. The results show that higher levels of materialism are statistically significantly associated with higher levels of compulsive buying. Ethnocentrism positively correlates with materialism but does not exhibit a direct relationship with compulsive buying. The mediating role of ethnocentrism between materialism and compulsive buying was not confirmed. The findings support theoretical assumptions about the influence of value orientations on problematic purchasing behavior. The study’s originality lies in integrating value-based and socio-cultural factors into a unified analytical model verified in the Slovak post-socialist context. From a theoretical perspective, the findings contribute to understanding how value orientations shape consumer behavior, while from a practical viewpoint, they provide implications for marketing strategies aimed at promoting responsible consumption and identifying risk groups prone to compulsive buying.

## 1 Introduction

In today’s consumer society, shaped by globalization and the dynamic development of markets, understanding the psychological and cultural foundations of purchasing behavior is becoming increasingly important. Among the most studied concepts in marketing and consumer behavior are materialism, consumer ethnocentrism, and compulsive buying — each representing specific values, attitudes, and behavioral patterns that influence consumer decision-making.

Materialism, as a value system that places high importance on acquiring and owning material goods, is repeatedly linked in the literature to reduced life satisfaction and increased susceptibility to impulsive or compulsive purchasing behavior. Compulsive buying, often referred to as shopping addiction, manifests as repeated, difficult-to-control urges to shop despite negative financial or psychological consequences for the individual. Previous research confirms a positive relationship between materialistic values and the tendency toward compulsive buying, particularly among younger age groups. Recent studies continue to support this association, emphasizing the mediating role of emotional regulation and self-control (Jain et al., 2024; Kyrios et al., 2020; Pahlevan Sharif et al., 2022).

In parallel, increasing attention has been devoted in recent years to consumer ethnocentrism — the belief that purchasing domestic products is right and morally preferable to buying foreign goods. Although ethnocentrism has traditionally been studied in relation to domestic brand preferences and loyalty to the country of origin, its potential link to compulsive or emotionally motivated purchasing behavior remains largely unexplored. More recent work highlights that ethnocentrism is not a static attitude but may interact with materialistic and identity-related motives (Chaturvedi et al., 2024; Lin et al., 2025; Czine et al., 2024).

While materialism, ethnocentrism, and compulsive buying have each been thoroughly examined individually in the literature, their interconnections have so far been only marginally investigated. Specifically, there is a lack of empirical research addressing how value-based orientations such as materialism combine with socio-cultural attitudes like ethnocentrism to shape excessive or problematic consumption (Nguyen and Pham, 2021; Cleveland et al., 2022). This absence of integrative studies is particularly evident in Central and Eastern Europe, where consumer behavior is influenced by historical transitions, local identity, and exposure to global consumer culture (Bryła and Domański, 2023; Aguilar-Rodríguez et al., 2025). Moreover, in the context of smaller Central European economies like Slovakia — shaped by specific socio-economic and cultural factors (e.g., post-communist transformation, the rapid rise of consumer capitalism after 1989, high sensitivity to local brands, and a historical distrust of foreign products) — such research is almost entirely absent. Understanding these patterns is essential for both academic and applied purposes, as they reflect not only individual value systems but also broader societal transformations in consumer identity formation.

This study therefore aims to fill this existing gap by examining whether and how consumer ethnocentrism may influence the relationship between materialistic values and the tendency toward compulsive buying in the Slovak context. By integrating these three concepts into a unified analytical model, the research contributes to a deeper understanding of the psychosocial mechanisms underlying risky purchasing behavior, while also providing practically applicable insights for marketing practice and the prevention of problematic consumption. The study is expected to add value by combining psychological (materialism), cultural (ethnocentrism), and behavioral (compulsive buying) dimensions into a single framework verified on a representative sample of Slovak consumers.

## 2 Theoretical backgrounds

Compulsive buying behavior, often referred to in the literature as shopping addiction, represents a specific form of dysfunctional consumer behavior. It is characterized by recurring, irresistible urges and desires to purchase various products. Essentially, it involves the consumer’s loss of impulse control over obsessive thoughts and feelings related to shopping. Compulsive behavior refers to unnecessary, uncontrollable, excessive, and repeated purchases of various products (Valence et al., 1988; Ridgway et al., 2008; Moon et al., 2022). Findings suggest that consumers with a tendency toward compulsive buying score significantly higher on the extraversion scale, meaning they are likely to appear passionate, sociable, decisive, active, adventurous, and optimistic (Gao et al., 2024).

Traditionally, compulsive buying has been primarily associated with economically developed Western countries, where the prevalence of this phenomenon ranges from approximately 2 to 16.4% of the population (Maccarrone-Eaglen and Schofield, 2017). In non-Western countries, although compulsive buying behavior is still in its early stages, reported prevalence rates are even higher, oscillating between 6 and 26.1% (Moon and Attiq, 2018).

Such behavior is increasingly recognized as a significant psychological and social issue (Kyrios et al., 2018; Müller et al., 2019). Moon et al. (2022) emphasize that compulsive buying implies serious individual, social, behavioral, financial, and economic consequences not only for the individual but also for society as a whole. The development of this pathological pattern of repeated, uncontrolled purchases is primarily conditioned by psychological factors (Tarka et al., 2022; Japutra et al., 2025).

The consequences of compulsive buying can be extensive, disrupting an individual’s financial situation and negatively affecting their mental health. Individuals with a history of compulsive buying demonstrate, for example, elevated levels of depression, lowered self-esteem, and a higher frequency of psychiatric issues, including mood disorders, eating disorders, and various addictions. They also exhibit anxiety disorders with obsessive thoughts and compulsive behaviors that trigger anxiety and disrupt their normal functioning (McElroy et al., 1994; Lawrence et al., 2014; Claes and Müller, 2017; Lam et al., 2018; Ünübol et al., 2022). Research has further shown that young consumers, in particular, display higher tendencies toward compulsive buying behavior (Adamczyk et al., 2020; Kyrios et al., 2020; Tarka et al., 2022).

Materialism is defined as a system of deeply rooted beliefs regarding the importance of material possessions in an individual’s life. Materialistically oriented consumers place ownership and the process of acquiring goods at the center of their lives, perceiving it as essential for achieving personal satisfaction and subjective well-being. Moreover, materialists tend to evaluate both their own success, and the success of others based on the quantity and quality of accumulated possessions. The construct of materialism is conceptualized through three fundamental dimensions: the centrality of material possessions in life, the perception of material goods as a means to happiness, and the definition of success through ownership of material goods (Richins and Dawson, 1992).

People with high materialistic values aspire to higher levels of social status and desire ownership of luxury products in an effort to achieve their primary life goals. For them, owning expensive items serves as a tool for presenting personal success, prestige, and social standing (Halliwell et al., 2005; Moon and Attiq, 2018). Nevertheless, in general, materialistic consumers exhibit lower levels of life satisfaction and happiness, suffer from increased anxiety and stress, experience identity crises, and often turn to the acquisition of specific product categories in an attempt to reduce dissonance in their self-concept (Kasser, 2016; Del Mar Alonso-Almeida et al., 2020).

In recent years, the dramatic rise of materialism has emerged as a social problem particularly among young consumers (Kasser et al., 2014). The motives behind materialistic attitudes can be traced to rapid urbanization, the high purchasing power of parents, and the role of young adults in family purchasing decisions (Tsang et al., 2014; Hultman et al., 2015). In marketing literature, materialism is often interpreted as a negative value, tending to have a negative impact on the subjective well-being and life satisfaction of young people (Froh et al., 2011), while showing a positive association with compulsive buying behavior (Manolis and Roberts, 2012; Lim et al., 2020).

Findings from previous research consistently indicate that young compulsive buyers score statistically significantly higher in the dimensions of materialism, such as importance, success, and happiness, while also exhibiting more intense symptoms of psychological distress, including anxiety, depression, obsessive-compulsive behavior, hostility, and somatization. In contrast, these individuals show significantly lower levels of self-esteem, life satisfaction, and optimism (Mueller et al., 2010; Villardefrancos and Otero-López, 2016). Research by Kyrios et al. (2020) pointed to strong endorsement of materialistic values among younger participants, with easier access to credit products and the revolution in online shopping potentially representing factors contributing to excessive buying problems in this age group (Choi et al., 2019).

Materialistic consumers perceive all their shopping activities as a means to acquire and own an excessive number of consumer products, with symbolic meaning for expressing their identity and emphasizing social status, which predisposes them to compulsive buying behavior. Therefore, it can be concluded that materialism has a naturally embedded relationship with compulsive buying behavior, one that is conditioned by the psychological and social aspects of consumption (Harnish et al., 2019; Tarka, 2020; Pahlevan Sharif et al., 2022).

Empirical findings from previous research also confirm that materialism represents a significant predictor of compulsive buying behavior (Islam et al., 2017; Khan et al., 2024). The fact that materialism is one of the key determinants influencing consumers’ purchasing decisions is further supported by the results of several other relevant studies (D’Astous, 1990; Mowen and Spears, 1999; Yurchisin and Johnson, 2004; Claes et al., 2016).

A significant phenomenon in the field of international economics and marketing is the tendency of consumers to show preference for domestically produced products and brands over foreign alternatives. This phenomenon, known as ethnocentrism, implies that consumer decisions are not determined solely by attributes such as price or quality, but that the country of origin of the product also plays a significant role (De Ruyter et al., 1998). Given the growing influence of globalization, consumer ethnocentrism may represent a relevant factor influencing the dynamics of contemporary markets.

Consumer ethnocentrism, in international marketing literature, is a concept adapted from the general concept of ethnocentrism and is defined as consumers’ beliefs about the appropriateness and morality of purchasing products made abroad (Shimp and Sharma, 1987). This concept thus refers to a preference for products that are culturally or geographically associated with one’s own country. Individuals with a higher level of ethnocentrism tend to favor domestic products over those originating from other regions, which subsequently affects their purchasing decision-making processes and consumer buying behavior.

Previous research has shown that the country of origin plays a key role in shaping consumer preferences for domestic products (Wanninayake and Chovancová, 2012; Ondrijová and Miško, 2024; Aguilar-Rodríguez et al., 2025). Specific findings in this area display some variability. For example, consumers in developing countries often exhibit lower levels of ethnocentrism and tend to prefer brands from economically more developed countries. In contrast, consumers in developed countries are generally characterized by a higher degree of ethnocentrism and show a stronger preference for domestic products.

An interesting finding is that, in some cases, consumers simultaneously hold strong ethnocentric feelings toward their home country while also maintaining positive perceptions of selected foreign products (Güneren and Öztüren, 2008; Shoham et al., 2017; Areiza-Padilla et al., 2021).

Consumer ethnocentrism reflects a preference for the values, products, and meanings associated with one’s own cultural group over those linked to other cultures. As a result, ethnocentrically oriented consumers perceive foreign groups negatively and avoid products associated with these cultures. Moreover, such consumers often hold the belief that products from their home country are of superior quality and reject purchasing from foreign suppliers and companies primarily out of loyalty to their own country (Bryła and Domański, 2023; Chaturvedi et al., 2024).

Consumers driven by ethnocentrism generally make purchasing decisions motivated by a desire for affirmation from their social groups, fueled by feelings of belonging and the need to adhere to shared values (Ben-Othmen and Kavouras, 2024; Aziz et al., 2025). These ethnocentric tendencies can significantly influence consumer purchasing behavior and intentions (Acikdilli et al., 2018; Dudziak et al., 2023; Lin et al., 2025).

Previous research studies have identified a positive correlation between high levels of consumer ethnocentrism and positive attitudes toward domestic products, which subsequently leads to stronger purchase intentions (Yildiz et al., 2018; Chakraborty et al., 2022; Hong et al., 2023; Czine et al., 2024).

The Slovak consumer environment is shaped by specific value orientations that have developed as a result of historical, cultural, and economic conditions. Byrska’s (2015) study shows that Slovak consumers hold distinct attitudes toward materialism, ownership, and consumption, reflecting their particular experiences with post-socialist transformation and globalization. These values influence not only preferences for domestic versus foreign products but also the degree of materialistic orientation and tendencies toward impulsive or compulsive buying.

In the Slovak context, consumer ethnocentrism has been examined by, for example, Vilčeková (2014), Sedláková et al. (2007), Táborecká-Petrovičová and Gibalová (2014), Gajdoš and Dziváková (2010), Saffu et al. (2010), Lesáková (2016), and Čvirik (2021). Compulsive consumption has been studied in Slovakia by Dzurová and Paholková (2016), Tomášová et al. (2024), and partially by Litavcová et al. (2015).

## 3 Theoretical framework

The conceptual model of this study is grounded in several complementary theoretical perspectives that explain the relationships between materialism, consumer ethnocentrism, and compulsive buying.

The theory of compensatory consumption behavior (Dittmar, 2005) posits that individuals use consumption as a means of coping with psychological distress or identity deficits. From this perspective, materialistic values serve as compensatory mechanisms through which individuals seek self-enhancement and emotional relief, often leading to compulsive purchasing behavior. Thus, materialism is expected to positively predict compulsive buying tendencies.

The Value–Attitude–Behavior (VAB) framework (Homer and Kahle, 1988) explains how internalized values shape attitudes, which in turn drive behavioral intentions. Within this framework, materialism represents a value orientation, consumer ethnocentrism reflects an attitudinal component, and compulsive buying behavior represents the behavioral outcome. Consequently, ethnocentrism may mediate the relationship between materialistic values and compulsive consumption, as individuals’ cultural and moral beliefs about domestic products influence how value orientations manifest in purchasing behavior.

Insights from Consumer Culture Theory (CCT) (Arnould and Thompson, 2007) help interpret the sociocultural dimension of the model. CCT emphasizes how consumption is shaped by identity construction, social norms, and global–local dynamics. In transitional economies such as Slovakia, historical and cultural legacies (e.g., post-socialist transformation, ambivalent attitudes toward foreign products) shape consumer ethnocentrism, potentially moderating or amplifying the effects of materialism on compulsive buying.

Together, these theoretical perspectives provide a multidimensional foundation for the proposed PLS-SEM model, where materialistic values are hypothesized to influence compulsive buying both directly and indirectly through consumer ethnocentrism. The model thereby integrates psychological, cultural, and behavioral determinants of excessive consumption within a unified explanatory framework.

The aim of the present research is to quantify the connections between materialism, consumer ethnocentrism, and compulsive buying in the context of Slovak consumers.

The research builds upon prior theoretical insights and empirical findings that suggest the existence of links between these constructs, while also highlighting the need for a more comprehensive, model-based understanding of their interactions.

Based on this foundation, hypotheses were formulated focusing on three levels of relationships:

1. The relationship between various dimensions of materialism (happiness, success, centrality of material possessions) and the three dimensions of consumer ethnocentrism (affective, cognitive, and behavioral);
2. The relationship between the dimensions of ethnocentrism and the tendency toward compulsive buying;
3. The relationship between selected sociodemographic characteristics and the degree of compulsive buying.

The formulated hypotheses are as follows:

- Examining the relationship between the “happiness” dimension (MVS Happiness) and the individual components of ethnocentrism (CES):

*H1a: There is a significant relationship between material values happiness and the affective dimension of consumer ethnocentrism.*

*H1b: There is a significant relationship between material values happiness and the cognitive dimension of consumer ethnocentrism.*

*H1c: There is a significant relationship between material values happiness and the behavioral dimension of consumer ethnocentrism.*

- They explore the relationship between the “success” dimension (MVS Success) and ethnocentrism (CES):

*H2a: There is a significant relationship between material values success and the affective dimension of consumer ethnocentrism.*

*H2b: There is a significant relationship between material values success and the cognitive dimension of consumer ethnocentrism.*

*H2c: There is a significant relationship between material values success and the behavioral dimension of consumer ethnocentrism.*

- They examine the relationship between the “centrality” dimension (MVS Centrality) and ethnocentrism (CES):

*H3a: There is a significant relationship between material values centrality and the affective dimension of consumer ethnocentrism.*

*H3b: There is a significant relationship between material values centrality and the cognitive dimension of consumer ethnocentrism.*

*H3c: There is a significant relationship between material values centrality and the behavioral dimension of consumer ethnocentrism.*

- They focus on the relationship between the individual dimensions of ethnocentrism (CES) and compulsive buying (CBB):

*H4a: There is a significant relationship between the affective dimension of consumer ethnocentrism and compulsive buying behavior.*

*H4b: There is a significant relationship between the cognitive dimension of consumer ethnocentrism and compulsive buying behavior.*

*H4c: There is a significant relationship between the behavioral dimension of consumer ethnocentrism and compulsive buying behavior.*

- It examines the relationship between selected identification variables (e.g., age, gender, economic activity, material security in childhood) and compulsive buying (CBB):

*H5: There is a significant relationship selected identification indicators and compulsive buying behavior*.

**Figure 1:**
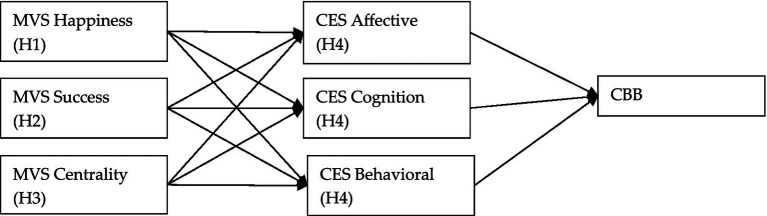
Schema of relationships in the PLS-SEM model.

The interrelationships between these hypotheses and latent variables are visualized in the following diagram (Figure 1), which presents the proposed PLS-SEM model. This model is based on the assumption that materialistic values influence ethnocentrism, which in turn (partially) mediates the effect on compulsive buying behavior.

Figure 1 illustrates the structure of the relationships between the individual constructs. Each arrow represents a hypothetical relationship between variables, where the arrows between MVS and CES reflect hypotheses H1–H3, the arrows between CES and CBB represent hypotheses H4a–H4c, and H5 denotes the additional influence of identification variables on the dependent variable. The model also assumes that the various dimensions of materialism and ethnocentrism may have different types of impacts on buying behavior — whether positive, negative, or none at all.

This conceptual schema provides a framework for the subsequent statistical analysis, whose aim is to test the validity of the assumed links and to evaluate to what extent the selected constructs can explain differences in compulsive buying among Slovak consumers.

## 4 Materials and methods

### 4.1 Material

The above assumptions were processed on a sample of Slovak respondents between March 13 and March 21, 2025. The sample consisted of 1,000 respondents who represent the population of the Slovak Republic over the age of 18 in terms of gender, age, education, marital status and economic status. The data were collected in cooperation with the professional research agency FOCUS (Bratislava, Slovakia) through its online respondent panel. The representativeness of the sample was ensured through a quota sampling technique based on key demographic parameters.

Table 1 provides the basic descriptive characteristics of the research sample. The distribution of respondents by gender, age, education, and other sociodemographic features indicates sufficient diversity, which increases the credibility of the findings. The sample predominantly includes young adults, which aligns with the research’s focus on the younger generation and their approach to sustainable behavior. The distribution of key variable values suggests balance and minimizes the risk of biased results. In addition to the identification characteristics presented, the research also works with the characteristic Type or focus of education (Economic (157, 15.7%), Humanities (135, 13.5%), Natural Sciences (61, 6.1%), Technical (305, 30.5%), General (342, 34.2%)), Size of place of residence (0–0.9 thousand (152, 15.2%), 1–1.9 thousand (145, 14.5%), 2–4.9 thousand (156, 15.6%), 5–19.9 thousand (163, 16.3%), 20–49.9 thousand (153, 15.3%), 50–99.9 thousand (93, 9.3%), over 100 thousand (138, 13.8%)), Personal net monthly income in EUR (up to 500 (192, 19.2%), 501–1,000 (396, 39.6%), 1,001–1,500 (263, 26.3%), 1,501–2000 (97, 9.7%), 2001–2,500 (25, 2.5%), over 2,500 (27, 2.7%)), Number of siblings (0 (91, 9.1%), 1 (353, 35.3%), 2 (294, 29.4%), 3 (155, 15.5%), 4 (64, 6.4%), 5 (32, 3.2%), 6 (11, 1.1%)), Mother’s highest attained education (primary (242, 24.2%), secondary without graduation exam (317, 31.7%), secondary with graduation exam (316, 31.6%), university degree I (22, 2.2%), university degree II (66, 6.6%), university degree III (8, 0.8%), do not know (29, 2.9%)), Father’s highest attained education (primary (164, 16.4%), secondary without graduation exam (395, 39.5%), secondary with graduation exam (278, 27.8%), university degree I (28, 2.8%), university degree II (69, 6.9%), university degree III (17, 1.7%), do not know (49, 4.9%)), During my childhood (I lived the whole time with both parents (812, 81.2%), my parents divorced/separated and then I lived only with my mother (112, 11.2%), my parents divorced/separated and then I lived only with my father (8, 0.8%), my parents divorced/separated and then I alternated between both (12, 1.2%), I lived only with my mother (43, 4.3%), I lived only with my father (4, 0.4%), I lived without parents (9, 0.9%)), During my childhood, in terms of material well-being, I felt (constant surplus of things I wanted (31, 3.1%), constant sufficiency of things I wanted (348, 34.8%), occasional lack of things I wanted (505, 50.5%), constant lack of things I wanted (116, 11.6%)).

**Table 1:** Sample characteristics.

Identification characteristic	Frequency	Percent
Gender		
Male	483	48.3%
Female	517	51.7%
Age		
18–24 years	85	8.5%
25–34 years	171	17.1%
35–44 years	202	20.2%
45–54 years	174	17.4%
55–64 years	159	15.9%
65 + years	209	20.9%
Education		
Primary education	57	5.7%
Secondary education without graduation exam	317	31.7%
Secondary education with graduation exam	383	38.3%
University degree I (Bachelor’s)	42	4.2%
University degree II (Master’s, Engineer, MD)	182	18.2%
University degree III (PhD)	19	1.9%
Marital status		
Single	256	25.6%
Married	469	46.9%
In long-term partnership (non-married)	146	14.6%
Divorced, currently without partner	80	8.0%
Widowed, currently without partner	49	4.9%
Economic status		
Student	59	5.9%
Employed	550	55.0%
Self-employed, entrepreneur	82	8.2%
Retired	205	20.5%
Unemployed	79	7.9%
On maternity/parental leave	25	2.5%

### 4.2 Conceptualization of survey

The first area processed in the analytical procedures was the Material Values Scale – MVS (Belk, 1985; Richins, 2004; Conejo et al., 2022; Ohno et al., 2022). The Material Values Scale (Richins, 2004) measures the extent to which individuals attribute importance to material possessions in their lives. It conceptualizes materialism as a three-dimensional construct, including the dimensions:

- Happiness – the belief that owning material things leads to life satisfaction,
- Success – the perception of possessions as indicators of life success,
- Centrality – the importance that material goods play in an individual’s life.

The scale items were evaluated as follows:

- *My life would be better if I owned certain things I do not have yet* (MVS_Happiness_1: Mean = 1.81, SD = 4.33)
- *The things I own say a lot about how well I am doing in life* (MVS_Success_1: Mean = 1.63, SD = 4.37)
- *I enjoy owning things that impress others* (MVS_Centrality_1: Mean = 1.77, SD = 3.23)
- *I admire people who own expensive homes, cars, and clothes* (MVS_Success_2: Mean = 1.88, SD = 3.12)
- *I like luxury in my life* (MVS_Centrality_2: Mean = 1.72, SD = 3.3)
- *I would be happier if I could afford to buy more things* (MVS_Happiness_2: Mean = 1.87, SD = 4.15)
- *Acquiring material possessions is among the most important achievements in my life* (MVS_Success_3: Mean = 1.69, SD = 2.84)
- *I feel that I have everything I need in life* (MVS_Happiness_3: Mean = 1.68, SD = 3.29)
- *I would be happier if I owned nicer things* (MVS_Happiness_4: Mean = 1.73, SD = 3.47)
- *The things I own are very important to me* (MVS_Centrality_3: Mean = 1.6, SD = 4.87)
- *I judge people by how many material things they own* (MVS_Success_4: Mean = 1.61, SD = 2.37)
- *Owning certain material possessions is a sign of success to me* (MVS_Success_5: Mean = 1.74, SD = 3.78)
- *I try to simplify my life by owning fewer things* (MVS_Centrality_4: Mean = 1.47, SD = 3.77)
- *Buying things gives me great satisfaction* (MVS_Centrality_5: Mean = 1.68, SD = 3.67)
- *It often bothers me that I cannot afford to buy everything I’d like* (MVS_Happiness_5: Mean = 1.81, SD = 4.11)

The next area processed in the analytical procedures was the Consumer Ethnocentrism Scale – CES (Sharma, 2015; Jiménez-Guerrero et al., 2020; Akbarov, 2022; Aguilar-Rodríguez et al., 2025). This scale assesses the degree to which individuals prefer domestically produced goods over foreign ones, based on moral or cultural beliefs. It is measured across three dimensions:

- Affective reaction – emotional attachment to domestic products (e.g., pride, admiration),
- Cognition bias – beliefs about the quality and superiority of domestic products,
- Behavioral preference – specific purchasing preferences and avoidance of foreign goods.

The scale items were evaluated as follows:

- *I love products and services from Slovakia* (CES_Affective_reaction_1: Mean = 1.51, SD = 5.13)
- *I take pride in products and services from Slovakia* (CES_Affective_reaction_2: Mean = 1.58, SD = 4.95)
- *I admire products and services from Slovakia* (CES_Affective_reaction_3: Mean = 1.54, SD = 4.93)
- *I feel attached to products and services from Slovakia* (CES_Affective_reaction_4: Mean = 1.71, SD = 4.46)
- *I hate products and services from foreign countries* (CES_Affective_reaction_5: Mean = 1.58, SD = 2.95)
- *I despise products and services from foreign countries* (CES_Affective_reaction_6: Mean = 1.61, SD = 2.85)
- *East or West, products and services from Slovakia are the best* (CES_Cognition_bias_1: Mean = 1.58, SD = 4.27)
- *Products from Slovakia are examples of top expertise* (CES_Cognition_bias_2: Mean = 1.56, SD = 4.35)
- *Service providers from Slovakia have the best work attitude* (CES_Cognition_bias_3: Mean = 1.6, SD = 4.04)
- *Products and services from foreign countries stand no chance against those from Slovakia* (CES_Cognition_bias_4: Mean = 1.71, SD = 3.43)
- *Slovakia has the hardest-working people in the industry* (CES_Cognition_bias_5: Mean = 1.7, SD = 4.68)
- *Service providers from Slovakia are more caring than those in any other foreign country* (CES_Cognition_bias_6: Mean = 1.62, SD = 3.91)
- *For me, Slovak products always come first* (CES_Behavioral_preference_1: Mean = 1.66, SD = 4.58)
- *If I have a choice, I prefer products and services from Slovakia* (CES_Behavioral_preference_2: Mean = 1.59, SD = 4.98)
- *I prefer to be served by Slovak service providers* (CES_Behavioral_preference_3: Mean = 1.59, SD = 4.64)
- *Whenever possible, I avoid buying products and services from foreign countries* (CES_Behavioral_preference_4: Mean = 1.71, SD = 3.65)
- *I often refuse to buy a product or service just because it’s from a foreign country* (CES_Behavioral_preference_5: Mean = 1.73, SD = 3.09)
- *I would rather not buy a product or service at all than buy one from a foreign country* (CES_Behavioral_preference_6: Mean = 1.71, SD = 3.02)
- These areas can be understood in the proposed model as independent variables.

The next area, understood as the dependent variable, was the domain of the Compulsive Buying: Original Measurement Scale (Valence et al., 1988; Lekavičienė et al., 2022). The original methodology serves to identify compulsive buying behavior, defined as repetitive, impulsive, and irresistible urges to shop, often associated with negative consequences. The scale assesses:

- Shopping impulses,
- Emotional regulation through shopping,
- Self-reflection and guilt after shopping.

The scale items were evaluated as follows:

- *When I have money, I cannot help but spend at least part or all of it* (CBB_1: Mean = 1.74, SD = 2.87)
- *I often behave impulsively when shopping* (CBB_2: Mean = 1.71, SD = 3.22)
- *For me, shopping is a way to cope with everyday stress and relax* (CBB_3: Mean = 1.73, SD = 2.84)
- *Sometimes I feel that something inside drives me to go shopping* (CBB_4: Mean = 1.81, SD = 2.87)
- *Sometimes I have a strong urge to buy something (clothes, books, etc.)* (CBB_5: Mean = 1.84, SD = 3.62)
- *Sometimes after buying a product, I felt a bit guilty because it seemed unreasonable* (CBB_6: Mean = 1.82, SD = 3.9)
- *Some things I buy, I do not show anyone out of fear they would see me as irrational (“unnecessary spending”)* (CBB_7: Mean = 1.79, SD = 2.8)
- *I often have an unexplained urge or sudden, spontaneous desire to go to a store and buy something* (CBB_8: Mean = 1.84, SD = 2.94)
- *As soon as I enter a shopping mall, I have an irresistible urge to go into a store and buy something* (CBB_9: Mean = 1.76, SD = 2.68)
- *I’m the kind of person who often responds to direct marketing offers* (e.g.*, phone, mail, or online*) (CBB_10: Mean = 1.63, SD = 2.54)
- *I’ve often bought a product I did not need even though I knew I had very little money* (CBB_11: Mean = 1.73, SD = 2.67)
- *I’m wasteful* (CBB_12: Mean = 1.63, SD = 2.46)
- *Sometimes I’ve thought: “If I could do it over again, I would…”* (CBB_13: Mean = 1.84, SD = 3.83)
- *During adolescence, I had enough money to occasionally buy things that pleased me* (CBB_14: Mean = 1.81, SD = 3.52)
- *Throughout adolescence, I was told how I should manage my money* (CBB_15: Mean = 1.8, SD = 4.36)
- *In case of financial problems, I know I could rely on someone to help me* (CBB_16: Mean = 1.92, SD = 4.31)

To ensure linguistic equivalence, the individual scales were translated from English into Slovak using automated translation. Subsequently, the translations were validated by a native speaker to minimize potential inaccuracies and ensure adequate semantic interpretation of the items in the Slovak context. The Slovak versions of the used scales are provided in the appendix.

Our research was conducted fully in accordance with the principles and best practices outlined in the European Code of Conduct for Research Integrity (ALLEA, 2024). We adhered to the principles of reliability, honesty, respect, and accountability throughout all phases of the research process. Specifically, our research was designed and carried out transparently and fairly, with respect to the research participants and full responsibility for its impact, as emphasized by the mentioned Code.

### 4.3 Methods

The analytical processes encompassed a wide range of methods, which can be divided into three main areas. In the first area, non-parametric difference tests were used to assess differences between categories of selected identification variables in compulsive buying. Specifically, the Kruskal-Wallis and Wilcoxon tests were applied. The aim of this study was built on analyzing relationships with the assumption of applying a regression model based on structural equations. A prerequisite for such an analysis is Confirmatory Factor Analysis (CFA). CFA was used to verify the factor structure of the applied scales in the Slovak context. After identifying items with low factor loadings (<0.5), these items were excluded, which improved the model’s validity indices (CFI, TLI, RMSEA, SRMR, AVE). To assess hypothetical relationships between latent variables in the model, Partial Least Squares Structural Equation Modeling (PLS-SEM) was applied. This approach is suitable for complex models with multiple latent variables and places fewer demands on distributional assumptions compared to traditional SEM. The results were evaluated based on bootstrapped estimates, t-statistics, and *p*-values. The analytical procedures were carried out using the R programming language (R Core Team, 2025).

## 5 Results

In the initial phase of the analytical process, we focused on examining the relationship between compulsive buying and selected identification variables. The first step was to verify the possibility of working on the aggregated CBB scale. The reliability of all 16 items of this scale was high (Cronbach’s *α* = 0.90), which allowed for their averaging and the creation of a new aggregated indicator called CBB mean.

Table 2 shows the results of the assessment of differences among selected identification characteristics. The results reveal significant differences in 5 out of the 12 tested cases. One of the strongest differences was identified in age categories (KW = 26.4, *p* value < 0.001), where a more detailed look at the results shows that the highest levels of compulsive buying are presented by the younger age groups (Mean: 18–24 years = 3.37, 25–34 years = 3.42, 35–44 years = 3.33, 45–54 years = 3.16, 55–64 years = 3.12, 65 + years = 2.98). Next, the highest average level of compulsive buying by marital status was identified in those in a permanent partnership without marriage (mean = 3.41), followed by single respondents (mean = 3.35), divorced (mean = 3.17), married (mean = 3.11), and finally respondents whose partner had died (mean = 3.03). From the perspective of economic status, the highest compulsive buying was identified among the unemployed (mean = 3.74), followed by those on maternity leave (mean = 3.71), students (mean = 3.25), unemployed (mean = 3.23), entrepreneurs (mean = 3.1), and finally pensioners (mean = 2.95). Regarding the number of siblings, the compulsive buying results are interesting, as the highest values were identified either at the minimum or maximum number of siblings (0 siblings - 3.32, 1 sibling - 3.28, 2 siblings - 3.21, 3 siblings - 3.13, 4 siblings - 2.76, 5 siblings - 3.48, 6 siblings - 3.36). The last area where significant differences were identified, concerning the perception of material security in childhood, showed an interesting result: the highest level of compulsive buying was found among respondents with a permanent surplus of material goods (mean = 3.90), followed by those with enough material goods (mean = 3.41), occasional lack of material goods (mean = 3.11), and finally those with a permanent lack of material goods (mean = 2.9).

**Table 2:** Difference test – compulsive buying across categories of selected identification variables.

Identification characteristic	Test	Statistic	*p* value
Gender	Wilcoxon	117,437	0.104
Age category	Kruskal-Wallis	26.4	**<0.001**
Education	Kruskal-Wallis	9.25	0.100
Household size	Kruskal-Wallis	10.6	0.102
Marital status	Kruskal-Wallis	19.9	**0.001**
Economic status	Kruskal-Wallis	34.9	**<0.001**
Income category	Kruskal-Wallis	9.48	0.091
Number of siblings	Kruskal-Wallis	16.1	**0.013**
Mother’s education	Kruskal-Wallis	10.2	0.115
Father’s education	Kruskal-Wallis	4.46	0.614
Family situation in childhood	Kruskal-Wallis	12.5	0.052
Material deprivation in childhood	Kruskal-Wallis	35	**<0.001**

Bold values indicate statistically significant results (*p* < 0.05).

In the subsequent analytical processes, confirmatory factor analysis (CFA) was applied, focusing on assessing the internal factor structure of the selected instruments. Although the instruments had been previously validated, it cannot be assumed that the validation conditions were identical to those in Slovakia. The initial CFA showed some deviations from expectations (*χ*^2^ = 8398.375, *p* value < 0.001, CFI = 0.766, TLI = 0.751, RMSEA = 0.081, SRMR = 0.102). These deviations were addressed by removing items with factor loadings below 0.5. Overall, 12 items were excluded from the analytical process (MVS Happiness 3, MVS Success 1, MVS Centrality 3, MVS Centrality 4, CES Affective Reaction 6, CES Affective Reaction 5, CES Behavioral Preference 6, CES Behavioral Preference 5, CBB 6, CBB 13, CBB 14, CBB 15, CBB 16). The removal was done progressively, with validity indicators checked at each step. After removal, the key validity indices reached generally acceptable values. The χ^2^ measure remained significant (*χ*^2^ = 3053.071, *p* value < 0.001), while the CFI index reached 0.903 and the TLI was 0.904. Both indices are at the borderline level of acceptability, where theory suggests a suitable threshold above 0.9. Similarly borderline values were observed for RMSEA (0.063) and SRMR (0.055). The so-called “rule of three” was complemented by the AVE characteristics (MVS Happiness = 0.588, MVS Success = 0.538, MVS Centrality = 0.510, CES Affective = 0.751, CES Cognition = 0.577, CES Behavioral = 0.626, CBB = 0.536). Based on this, it can be concluded that reliability is above 0.5 in all cases, meaning the level is acceptable.

Table 3 summarizes the results of the Partial Least Squares Structural Equation Modeling (PLS-SEM) and provides an overview of the tested hypotheses. The table structure includes columns for hypothesis labels, definitions of the examined relationships between variables, the original estimated coefficient (Original Est), the average bootstrap estimate (Bootstrap Mean Est), the standard deviation of the bootstrap sample (Bootstrap SD), the t-test statistic (T Stat), and the *p*-value (*p* Value) for testing the statistical significance of each relationship.

**Table 3:** Outputs of the PLS SEM model.

Hyp	Relations	Original Est	Bootstrap Mean Est	Bootstrap SD	T Stat	*p* value
H1a	MVS_Happiness → CES_Affective	−0.054	−0.050	0.067	−0.809	0.418
H1b	MVS_Happiness → CES_Cognition	0.013	0.016	0.053	0.242	0.809
H1c	MVS_Happiness → CES_Behavioral	−0.023	−0.020	0.065	−0.356	0.722
H2a	MVS_Success → CES_Affective	−0.103	−0.025	0.109	−0.945	0.345
H2b	MVS_Success → CES_Cognition	0.239	0.238	0.053	4.546	**<0.001**
H2c	MVS_Success → CES_Behavioral	0.190	0.184	0.083	2.300	**0.021**
H3a	MVS_Centrality → CES_Affective	0.113	0.080	0.081	1.403	0.160
H3b	MVS_Centrality → CES_Cognition	−0.041	−0.038	0.073	−0.556	0.578
H3c	MVS_Centrality → CES_Behavioral	−0.019	−0.017	0.081	−0.232	0.816
H4a	CETS_Affective → CBB	−0.235	−0.170	0.104	−2.268	**0.023**
H4b	CETS_Cognition → CBB	0.260	0.264	0.054	4.829	**<0.001**
H4c	CETS_Behavioral → CBB	0.089	0.075	0.076	1.164	0.244

Bold values indicate statistically significant results (*p* < 0.05).

Out of the total 12 tested relationships, 4 statistically significant relationships were identified based on the PLS-SEM results, with significance assessed at the *p* < 0.05 level. Specifically, significant positive and strong effects were confirmed between MVS Success and CES Cognition (H2b) (*β* = 0.239; *p* < 0.001), and between MVS Success and CES Behavioral (H2c) (*β* = 0.190; *p* = 0.021), indicating a positive, moderately strong association. A statistically significant negative effect was found between CES Affective and CBB (H4a) (*β* = −0.235; *p* = 0.023). Lastly, a significant positive effect of CES Cognition on CBB (H4b) was identified (*β* = 0.260; *p* < 0.001).

Based on these results, we recommend accepting hypotheses H2b, H2c, H4a, and H4b. These hypotheses highlight important links between the materialistic value component “Success” and the cognitive and behavioral dimensions of consumer ethnocentrism, as well as the relationships between the affective and cognitive dimensions of ethnocentrism and compulsive buying behavior.

Conversely, all other hypotheses (H1a, H1b, H1c, H2a, H3a, H3b, H3c, H4c) were not supported by statistically significant results and are therefore recommended for rejection.

**Figure 2:**
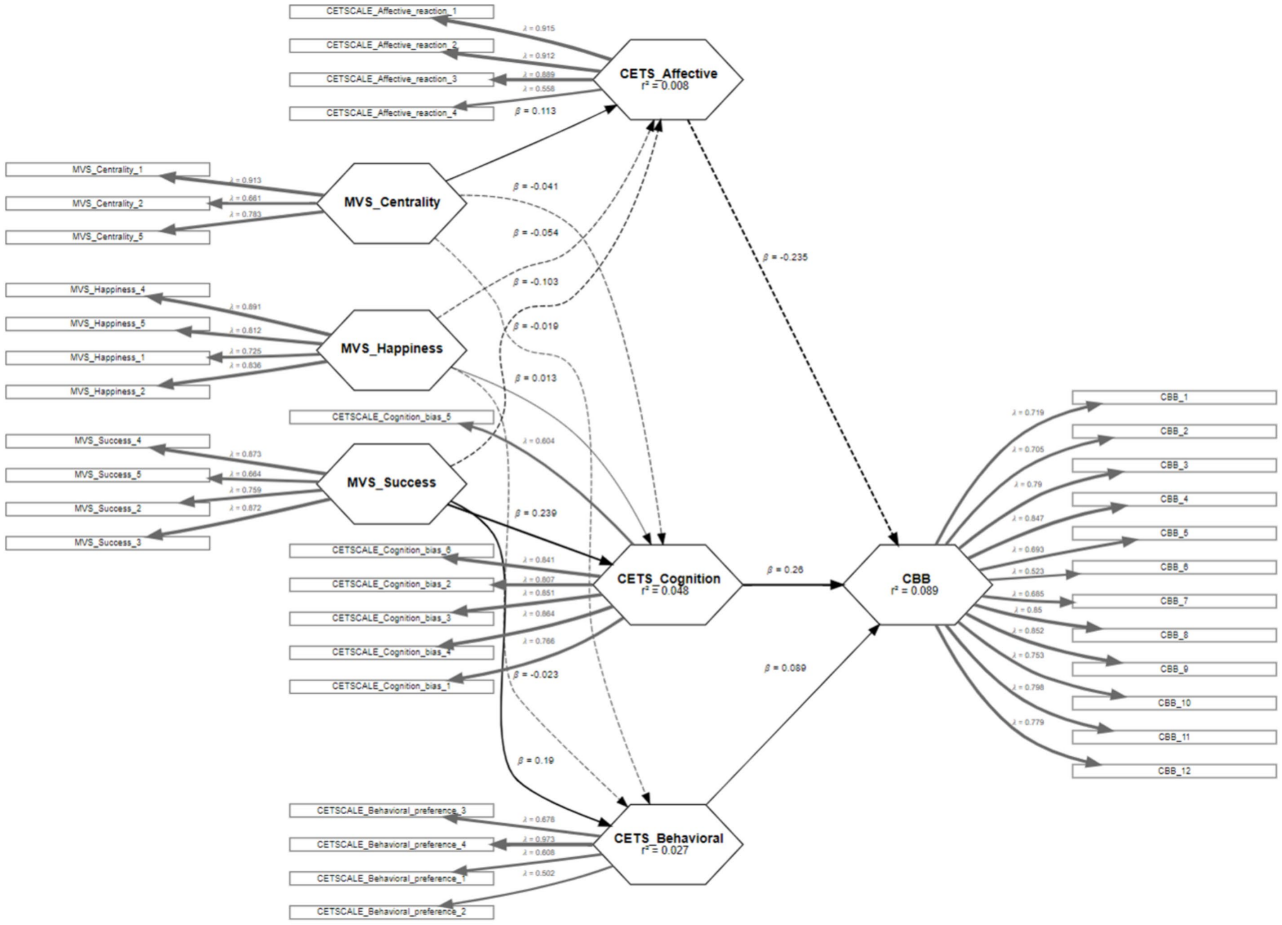
Relationship diagram.

The diagram (Figure 2) illustrates the relationships between materialistic values (MVS), consumer ethnocentrism (CES), and compulsive buying behavior (CBB). Solid arrows indicate positive correlations, while dashed arrows represent negative correlations. The strongest positive relationship is between MVS Success and CES Cognition (*β* = 0.239). The diagram also shows the determination coefficients (R^2^), which are generally low, suggesting that the model does not fully explain the variability of the phenomena and that other constructs likely exist to better predict compulsive buying behavior.

## 6 Discussion

The aim of this study was to explore the relationships between materialistic values, ethnocentrism, and compulsive buying behavior (CBB) in the Slovak context. The results were analyzed using Partial Least Squares Structural Equation Modeling (PLS-SEM) and supplemented by difference testing across selected respondent categories.

The study suggests that materialism is a significant risk factor for compulsive buying. The findings showed that higher materialistic values correlate with increased compulsive buying tendencies. This is supported by a meta-analysis by Nguyen and Pham (2021), which found a strong relationship between materialism and compulsive buying, highlighting that individuals with high materialistic values are more likely to engage in compulsive purchasing behavior.

One of the main findings of this study was that the materialistic value “Success” (MVS Success) has a positive relationship with the cognitive and behavioral dimensions of ethnocentrism. This result is fully consistent with findings by Cleveland et al. (2022), who in a multicultural study confirmed that materialistically oriented consumers may also be more inclined toward domestic brands when they perceive consumption as a display of social status or identity. In the Slovak research context, this phenomenon is particularly evident in the relationship between materialism and the rationalized (cognitive) preference for domestic products, which supports hypotheses H2b and H2c. These results also confirm conclusions from previous research showing that materialistic values are not just individual attitudes but manifest in value-driven purchasing behavior (Lim et al., 2020; Islam et al., 2017). Research by Pahlevan Sharif et al. (2022) indicated that materialism is reinforced by social comparison and media pressure, which can lead to behavioral patterns of overspending.

On the other hand, the dimensions of materialism oriented toward happiness and centrality of material possessions in life (MVS Happiness and MVS Centrality) did not show statistically significant relationships with ethnocentrism. This may suggest that the emotional aspects of desire for material ownership do not automatically lead to a preference for domestic products. The partial mismatch with the proposed model may be related to what Tarka et al. (2022) describe as a different motivational structure of hedonic buying – that is, materialism does not necessarily lead to local patriotism in consumption behavior but rather to individualism and a desire for personal pleasure. This interpretation is particularly important in relation to younger consumers, who, according to this study, exhibit the highest levels of compulsive buying.

Another important finding is the significant relationship between ethnocentrism and compulsive buying. Within hypotheses H4a and H4b, it was confirmed that ethnocentrism, especially its cognitive (CES Cognition) and affective component (CES Affective), can influence compulsive buying behavior. Although one might assume that a preference for domestic products would protect against impulsive behavior, the results suggest the opposite. Similarly, studies by Lin et al. (2025) and Hong et al. (2023) identified that a strong belief in the superiority of domestic products can lead to irrational and impulsive behavior when the consumer is exposed to a domestic-origin stimulus. However, the negative relationship between affective ethnocentrism and compulsive buying (H4a) may indicate that positive emotional experiences related to domestic brands can reduce the need for compensatory purchasing, which aligns with findings on emotional regulation in buying behavior (Gao et al., 2024). These results suggest that while rational belief in the superiority of domestic products may lead to more frequent (and potentially impulsive) buying behavior, a strong emotional attachment to domestic products can act as a regulatory factor. This paradoxical relationship corresponds with findings by Jain et al. (2024), who highlight the role of emotional processes and self-regulation in the development of compulsive buying. A similar study by Moldes et al. (2025) examined how ethnocentric attitudes may affect consumer behavior, especially in developing markets where local identity may conflict with global consumer culture, potentially leading to compulsive buying as a means of asserting identity.

Statistically significant differences in the level of CBB among various sociodemographic groups were also consistent with international trends. The highest compulsive buying scores were exhibited by younger respondents, the unemployed, people on parental leave, and those who experienced material abundance in childhood. These findings are consistent with the literature (Tarka et al., 2022; Adamczyk et al., 2020), which points to greater vulnerability of certain sociodemographic groups to buying behavior as a form of compensation for psychological or social deficits. This is supported by recent research suggesting that younger populations are more susceptible to materialistic influences and compulsive buying, often driven by social status and peer comparison (Roberts et al., 2003). Regarding the higher CBB rates among the unemployed, the results align with studies indicating economic instability as a factor contributing to compulsive buying, especially among unemployed individuals who may seek material goods as a coping mechanism (Antinienė et al., 2021). The results also correspond with findings by Wang and Zhai (2022), who demonstrated in an intercultural study that long-term orientation and modest upbringing values can act as protective factors against CBB. In our research, respondents with material abundance in childhood exhibited higher levels of CBB, suggesting that early social experience may shape excessive consumer behavior. Similar conclusions were drawn by Moulding et al. (2017), who suggested that early exposure to material wealth may reinforce materialistic values, leading to compulsive buying behavior in adulthood. This suggests that not deficit, but abundance may lead to the formation of habits associated with consumer self-regulation, supported also by findings from Kasser et al. (2014). Opposite results were found by Islam et al. (2017), who discovered that material deprivation in childhood may lead to higher materialism and subsequently to compulsive buying behavior. Contrary to some other studies (e.g., Tarka et al., 2022; Miltenberger et al., 2003), parental education did not prove to be a significant predictor of compulsive buying. Das and Mukherjee (2020) also suggest that parental influence may shape consumer behavior, although the effect can vary across different cultural contexts and economic environments. This may be a specificity of the Slovak environment, where social mobility is not strictly tied to parents’ formal education.

The results of this study thus highlight the complexity of consumer behavior, which is influenced not only by values and attitudes but also by life experiences and current socio-economic status. The combination of materialism and rational ethnocentrism appears to be a risk factor for compulsive buying, whereas an emotional attachment to domestic products may act protectively. From a theoretical perspective, the findings suggest the need to expand existing models with additional variables such as digital literacy, debt levels, impulse processing, or subjective stress levels (Japutra et al., 2025; Choi et al., 2019). From a practical standpoint, the study can be utilized in design campaigns focused on responsible consumption, personalized marketing, or interventions targeted at at-risk groups.

The main contribution of this study lies in linking previously mostly separately examined concepts of materialism, ethnocentrism, and compulsive buying into a unified model, thereby broadening the current understanding of the mechanisms of consumer behavior. Previous research has addressed the relationships between materialism and compulsive buying (Islam et al., 2017; Lim et al., 2020) or between ethnocentrism and purchasing preferences (Hong et al., 2023; Lin et al., 2025), but an analysis of their combined effects is lacking. By verifying these relationships in the specific postsocialist context of Slovakia, the study additionally contributes to expanding knowledge predominantly derived from Western contexts (Antinienė et al., 2021; Kasser et al., 2014), allowing for the identification of cultural specifics that may influence the development of compulsive buying behavior.

## 7 Conclusion

The initial steps of the analytical processes focused on identifying differences in compulsive buying across categories of selected demographic variables. Higher levels of compulsive buying were observed among younger respondents, individuals in stable partnerships without marriage, the unemployed, and those on maternity leave. An interesting finding was that the highest scores appeared among respondents with both minimal and maximal numbers of siblings. Respondents who experienced a consistent material surplus in childhood also demonstrated a greater tendency toward compulsive buying. These findings suggest a complex interaction of social and economic factors.

Subsequently, a PLS-SEM regression model was applied. The results of this model show that only some of the hypothesized relationships were confirmed, with statistically significant effects mainly linking materialistic values and consumer ethnocentrism, as well as certain connections between ethnocentrism and compulsive buying behavior. This partial support for the originally formulated hypotheses is scientifically interesting because it indicates that consumer values and attitudes — although often considered universal predictors of behavior — do not fully explain all expected relationships in this case.

Particularly, the confirmation of the influence of the materialistic value “success” on both the cognitive and behavioral components of ethnocentrism suggests that perceiving success as a value may significantly affect attitudes toward domestic products and preferences for local brands. Similarly, the identified link between ethnocentrism and compulsive buying behavior indicates that purchasing decisions may be partially influenced by emotional and value-based factors, opening avenues for further research on consumer behavior patterns.

The results are thus not entirely unexpected, but the division between confirmed and unconfirmed hypotheses signals that the relationships between values, attitudes, and behavior are more complex than a linear model suggests. Practically, this means that consumer behavior cannot be straightforwardly explained by values or attitudes alone, as additional factors—such as situational context or cultural differences—likely influence decision-making. This type of outcome is common in social science research; however, the fact that some relationships were confirmed strengthens the model’s credibility and indicates its practical applicability in marketing research and further theoretical development.

While the study’s findings provide important insights into the relationships among materialistic values, consumer ethnocentrism, and compulsive buying, certain methodological limitations should be noted that may affect their interpretation. The first limitation is the cross-sectional nature of the research, which allows for identifying associations between variables but does not permit causal inferences. Therefore, the results should be interpreted as correlations rather than evidence of causality. Nonetheless, these findings are considered relevant as they build upon existing literature and extend understanding of the constructs studied.

Another limitation is the relatively low explained variance of the dependent variable (compulsive buying), indicating that the model captures only part of the factors influencing this phenomenon. However, this result aligns with findings from behavioral and psychological sciences, where consumer behavior is known to be influenced by a wide array of variables. From a theoretical standpoint, the relationships identified remain statistically significant and meaningful in interpretation.

Future research plans include the use of more complex analytical approaches to enable a more precise assessment of causal relationships. Additionally, the model will be extended to incorporate other relevant variables that could increase explained variance and improve understanding of predictors of compulsive buying behavior.

## Data Availability

The datasets presented in this article are not readily available because the dataset is not publicly available due to ethical and privacy restrictions. The data contain information that could potentially be sensitive, and although collected anonymously, the terms of participation did not include consent for public data sharing. Requests to access the datasets should be directed to ivana.ondrijova@unipo.sk.
